# Research Productivity Among Applicants Matching to Top Pediatrics Residency Programs in the Post-pass/Fail Step One Era

**DOI:** 10.7759/cureus.107366

**Published:** 2026-04-19

**Authors:** Jonathan Gulkarov, Nicholas Minasian, Elana Eisenreich, David Kakzanov, Liel Kalen, Daniel Murdahayev, Daniel Yusupov, Boris Mulod, Matthew Neryaev, Mark M Shimanov

**Affiliations:** 1 Biology, St. John's University, Queens, USA; 2 Medicine, State University of New York Downstate Health Sciences University, Brooklyn, USA; 3 Cardiology, City University of New York School of Medicine, New York, USA; 4 Internal Medicine, State University of New York Downstate Health Sciences University, Brooklyn, USA; 5 Biology, Hofstra University, Hempstead, USA

**Keywords:** nrmp match, pass/fail usmle, pediatric residents, research productivity, residency program selection, united states medical licensing examination, united states medical licensing examination scoring

## Abstract

Background

The transition to pass/fail scoring for the United States Medical Licensing Examination (USMLE) Step one has shifted residency selection toward alternative metrics, including research productivity. While scholarship expectations are well-characterized in competitive surgical specialties, less is known about research output among pediatrics applicants.

Objective

To characterize research productivity of applicants successfully matching into the Doximity-ranked top 10 pediatric residency programs and examine differences by institutional National Institutes of Health (NIH) funding rank and gender.

Methods

All 450 students matching into the Doximity-ranked top 10 pediatric programs in 2024 were identified using Doximity rankings and program rosters. Publications through September 27, 2024, were catalogued using Google Scholar and classified by type, authorship position, pediatrics relevance, and journal h-5 index. Applicants were stratified by medical school NIH funding rank (Top 40 vs 41-150) and gender. Welch's t-tests and Chi-square analyses compared publication metrics between groups.

Results

Mean publications were 2.3 ± 4.7 (median=1.0); 180/450 (40.0%) of matched applicants had zero publications. Clinical research was most common (449/1029, 43.6%), followed by basic science (269/1029, 26.1%). Students from NIH Top-40 institutions were more likely to have publications (113/159, 71.1% vs 157/291, 54.0%, p<0.001), though publication quantity and authorship metrics did not differ by institutional rank or gender among those with research experience. Research output increased progressively through medical school, particularly for pediatrics-related and first-author publications.

Conclusions

Successful applicants to elite pediatrics programs demonstrate heterogeneous research backgrounds, with substantial proportions matching without publications. While institutional funding influences research access, productivity metrics remain comparable across institutions and genders once students engage in scholarship, supporting holistic residency evaluation practices.

## Introduction

Pediatrics is widely recognized as a less competitive specialty compared to many others, maintaining a match rate of 97-98% since 2017 [1], with the exception of 2024 when there was a six percentage-point decline in the match rate [2]. This notable drop may reflect systemic issues such as inadequate training capacity and comparatively low compensation for pediatricians, which have been identified as key deterrents for new U.S. MD graduates considering the specialty [3]. The financial burden is particularly acute, given that physicians who graduate from medical school carry anywhere from $50,000 to 100,000 in debt, making lower-compensated specialties like pediatrics less financially viable despite the student's interest in child health [4]. One major reason for the historically high match rate is the large number of available positions relative to the applicant pool. For example, in 2024, there were 3,078 Categorical pediatrics positions offered, yet only 2,827 were filled, representing a fill rate of 91.85% [2]. In contrast, surgical specialties notably have far fewer positions available but attract a greater number of applicants than there are spots, creating a much lower match rate and higher competition. As a result, these programs typically fill nearly all of their positions, with total fill rates approaching 99-100% [5].

The recent decline in pediatrics match rates raises important questions about the evolving landscape of residency selection and applicant competitiveness within the specialty. Despite pediatrics' reputation for accessibility, the 2024 Match data suggest that certain positions may be becoming more selective, or that qualified applicants are increasingly choosing alternative career paths. The transition to pass/fail scoring for the United States Medical Licensing Examination (USMLE) Step one in 2022 has fundamentally altered the residency application landscape across all specialties, prompting program directors to place greater emphasis on alternative metrics for applicant evaluation [6]. Research productivity, clinical experience, letters of recommendation, and Step two Clinical Knowledge (CK) scores have emerged as increasingly important distinguishing factors in applicant portfolios [7].

Understanding the research output of successfully matched pediatrics applicants is particularly relevant in this changing environment. While previous studies have characterized research productivity among applicants to highly competitive specialties such as plastic surgery, orthopedic surgery, and otolaryngology [8], less attention has been devoted to examining these trends in pediatrics. Given that pediatrics maintains high overall match rates while top-tier programs remain highly selective, it is essential to characterize current research expectations and determine whether trends observed in more competitive specialties are beginning to appear among applicants to elite pediatrics programs. To our knowledge, no study has specifically characterized research productivity among applicants successfully matched to elite pediatrics programs in the post-pass/fail Step one era. This study addresses that gap by characterizing publication patterns, authorship metrics, and research trends among matched applicants to the Doximity-ranked top 10 pediatric residency programs in the 2024 Match.

## Materials and methods

Utilizing the 2023-2024 Doximity Residency Navigator [9], 10 of the nation's most reputable pediatric programs were selected. To confirm matriculants' enrollment in these programs, social media platforms such as Instagram and Twitter were employed [10,11], along with program websites. Demographic information, including sex, was recorded for each participant. Each matriculant was evaluated for the number of peer-reviewed publications published before September 25, 2024. Google Scholar was utilized to ensure that publications were accurately attributed to their corresponding individuals [12]. Study data were collected and managed using REDCap electronic data capture tools hosted at the State University of New York (SUNY) Downstate Health Sciences University College of Medicine [13,14]. This study used publicly available, de-identified data and did not involve human subjects. Therefore, institutional review board approval and informed consent were not required. The determination of whether a publication was relevant to pediatrics was made by reviewing both the journal of publication and the article's abstract.

Publications were classified into the following categories: clinical research, basic science research, case reports, author replies, and review articles. Articles that did not align with any of these categories were labeled as "other." Clinical research was defined as studies involving any form of patient-related analysis. Basic science research encompassed both laboratory-based experiments and translational investigations. Case reports referred to analyses focusing on a single patient. Review articles included studies that synthesized previously published literature to guide the analysis (e.g., systematic reviews, meta-analyses). For each publication, the journal's h-5 index and the total citation count were recorded using Google Scholar [15], and the authorship position of the matriculant was noted. 

A binomial test was performed (test proportion of 0.50) to determine if there were significantly more male or female applicants accepted into the Doximity-ranked top 10 pediatric residency programs. Publication types were categorized and graphed by pediatrics-related and non-pediatrics-related publications. Trends in total publications, first-author publications, pediatrics-related, non-pediatrics-related, basic science, and clinical publications were analyzed over time. The timeline was anchored to September 27, 2023, the date when programs were permitted to begin application review. Publications were categorized by medical school year based on the following date ranges: pre-matriculation (before September 28, 2020), first year (September 28, 2020 to September 27, 2021), second year (September 28, 2021 to September 27, 2022), third year (September 28, 2022 to September 27, 2023), and fourth year (September 28, 2023 to September 27, 2024). The fourth-year interval was not pro-rated to the Match date (March 15, 2024) and instead included all publications through September 27, 2024, thereby capturing work completed during the post-Match period of the final academic year. Notably, publications appearing between Match Day and our data collection date could not have influenced match outcomes, though they were included to capture the full scope of research productivity during medical school. Descriptive statistics were calculated and continuous variables were reported as mean ± standard deviation. Applicants were stratified by medical school National Institutes of Health (NIH) funding rank into the top 40 (NIH T40) and NIH 41-150 groups. Welch’s t-tests were used to compare publication metrics between groups, including total publications, first-author publications, top-two and top-five author publications, pediatrics-related publications, basic science publications, clinical publications, and average journal h-5 index ranking. All analyses were repeated to assess gender-related differences across the entire cohort. Research output, defined as the presence of at least one publication, was analyzed as a categorical outcome. Differences in research output were compared across NIH funding tiers (T40 vs. 41-150) and sex (male subjects vs. female subjects) using the Pearson Chi-square test. A two-sided p<0.05 was considered statistically significant. All statistical analyses were performed using IBM SPSS Statistics for Windows, Version 25 (Released 2022; IBM Corp., Armonk, New York, United States). 

## Results

**Figure 1:**
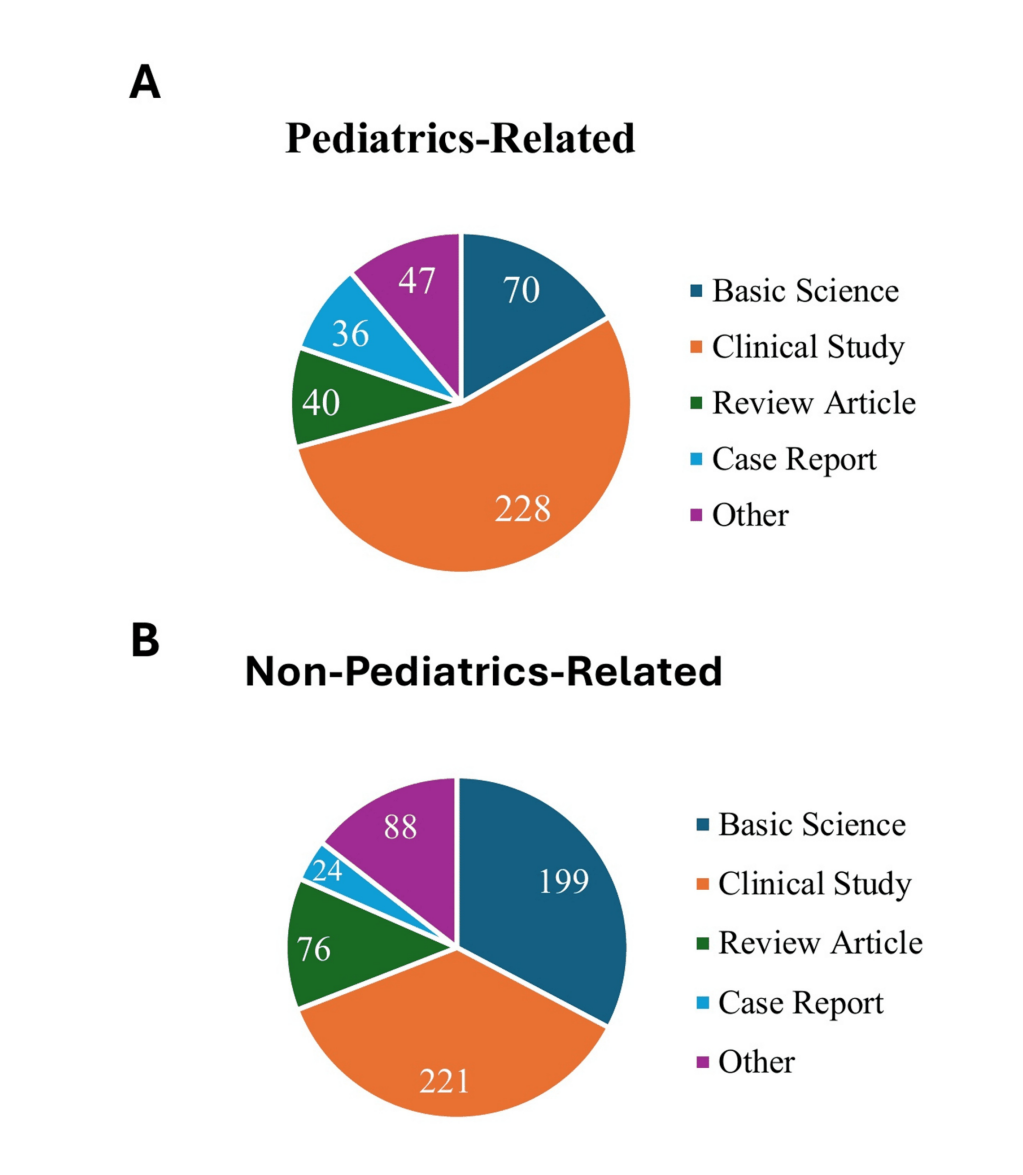
Distribution of journal types among pediatrics-related and non-pediatrics-related publications (A) Breakdown of basic science, clinical studies, review articles, case reports, and other journal types among pediatrics-related publications; (B) Breakdown of basic science, clinical studies, review articles, case reports, and other journal types among non-pediatrics-related publications.

All 450 students matching into the top 10 pediatrics residency programs in the 2024 Match were included in this study. The majority were female subjects (351/450, 78.0%), representing a statistically significant proportion of the applicant pool (p=0.046). Stratification by article type showed that clinical studies were the most common publication type, accounting for 449/1029 (43.6%) of all publications, followed by basic science at 269/1029 (26.1%) and review articles at 116/1029 (11.3%) (Figure 1).

This study found that there was no significant difference between the number of pediatrics and non-pediatrics related papers (p=0.887), suggesting that these students’ research interests and opportunities spanned a few medical disciplines (Table 1).

**Table 1:** Comparison of journal-type metrics by NIH T40 vs. T41–150 institutions and applicant sex Descriptive and inferential statistics comparing the total number of publications, journal types, and mean h-5 impact factor between applicants from the National Institutes of Health (NIH) T40 versus T41–150 institutions and between male and female applicants. The cohort of applicants with publications included only those with at least one publication, while the cohort of applicants including those without publications is inclusive of all applicants in the study. NIH: National Institutes of Health

Publication metrics	NIH T40	NIH T41-150	p-value	Male applicant	Female applicant	p-value
Total publications	2.7 ± 4.3	2.1 ± 4.9	0.17	2.9 ± 4.6	2.2 ± 4.7	0.14
First authorships	0.8 ± 1.4	0.6 ± 1.5	0.96	0.8 ± 1.6	0.6 ± 1.4	0.33
Pediatrics-related publications	1.0 ± 1.8	0.9 ± 1.9	0.27	1.0 ± 1.9	0.9 ± 1.9	0.86
Basic science publications	0.8 ± 2.3	0.5 ± 1.3	0.26	0.9 ± 2.2	0.5 ± 1.6	0.18
Clinical studies	1.1 ± 2.2	0.9 ± 3.5	0.74	1.0 ± 2.5	1.0 ± 3.2	0.77
h-5 impact index mean (with publications)	78.2 ± 45.2	67.4 ± 40.0	0.044	83.6 ± 53.6	68.9 ± 38.2	0.051
h-5 impact index mean (Including those without publications)	55.6 ± 52.1	35.8 ± 44.4	<0.001	52.3 ± 58.7	40.4 ± 44.8	0.068

**Figure 2:**
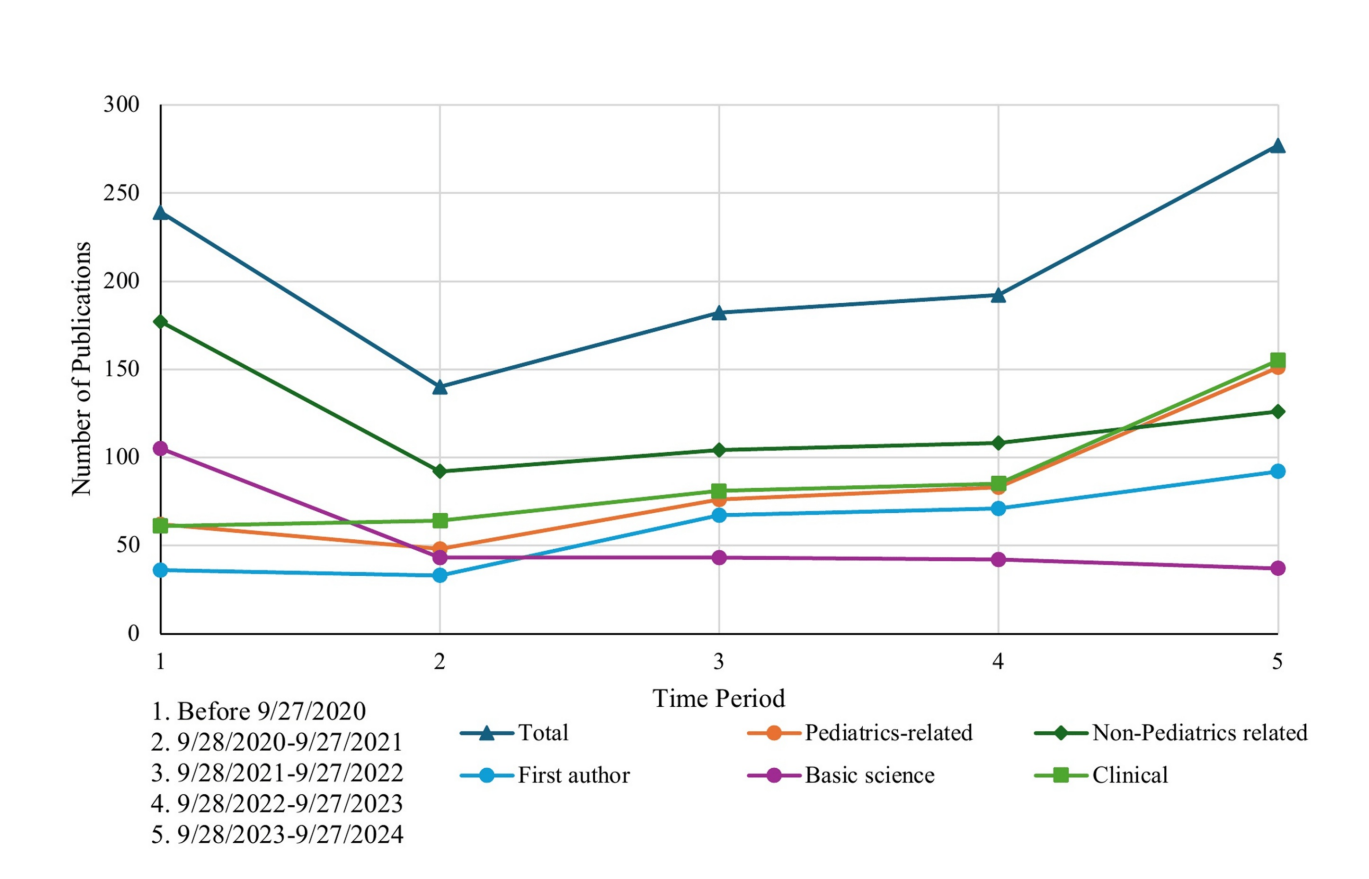
Number of publications, categorized by the academic year of medical school Distribution of applicant publications according to the academic year of medical school in which the work was published, demonstrating trends in research productivity over the course of medical training.

There was also a lower proportion of first authorships, which did climb as medical school progressed, but only accounted for 298/1029 (29.0%) of the total publications. We found that 135/1029 (13.1%) of papers fell into the “Other” category and case reports comprised only around 60/1029 (5.8%) of the total publications. The number of pediatrics-related first-author publications and clinical publications increased over time as students progressed through their medical education (Figure 2).

Overall, the students averaged 2.3 ± 4.7 publications (Median=1.0). 180/450 (40.0%) students had zero publications, while 270/450 (60.0%) students had at least one publication. These 270 students published 1,029 papers before September 27, 2024, across more than 140 journals. Descriptive data broken down by percentile rankings for various publication parameters are displayed in Table 2.

**Table 2:** Percentile rankings of the applicants' publication metrics Percentile rankings for key publication metrics among applicants, including measures of research productivity and journal impact. These metrics include the number of publications, first authorships, top two authorships, top five authorships, basic science articles, clinical studies, pediatrics-related articles, and the h-5 journal impact factor. The cohort of applicants with publications includes only those with at least one publication, while the cohort of applicants, including those without publications, is inclusive of all applicants in the study. SD: Standard deviation

Metric	Mean ± SD with publications	Mean ± SD, including those without publications	90th percentile	80th percentile	70th percentile	60th percentile	50th percentile	40th percentile	30th percentile	20th percentile	10th percentile
Number of publications	3.9 ± 5.5	2.3 ± 4.7	6	3.8	2	1	1	0.4	0	0	0
1st authorships	1.1 ± 1.7	0.7 ± 1.5	2	1	1	0	0	0	0	0	0
Top 2 authorships	1.8 ± 2.5	1.1 ± 2.1	3	2	1	1	0	0	0	0	0
Top 5 authorships	3.0 ± 4.1	1.8 ± 3.5	5	3	2	1	1	0	0	0	0
Basic science publications	1.0 ± 2.2	0.6 ± 1.7	2	1	0	0	0	0	0	0	0
Clinical studies	1.7 ± 3.9	1.0 ± 3.1	2.8	1	1	0	0	0	0	0	0
Pediatrics-related publications	1.6 ± 2.2	0.9 ± 1.9	3	1	1	0	0	0	0	0	0
h-5 journal impact factor	71.9 ± 42.4	42.7 ± 48.1	107.7	80.6	59.6	47	34	0	0	0	0

Among all applicants, the mean number of publications for those with at least one publication was 3.9 ± 5.5, while including students without publications, the mean decreased to 2.3 ± 4.7. Similarly, the mean number of first authorships, top two authorships, and top five authorships were higher when considering only students with publications (1.1 ± 1.7, 1.8 ± 2.5, and 3.0 ± 4.1, respectively) compared with the full cohort (0.7 ± 1.5, 1.1 ± 2.1, and 1.8 ± 3.5). When stratified by research type, applicants had on an average 1.0 ± 2.2 basic science publications, 1.7 ± 3.9 clinical publications, and 1.6 ± 2.2 pediatrics-related publications among those with at least one publication, with corresponding lower means when including students without publications. The h-5 index of journals in which applicants published had a mean of 71.9 ± 42.4 among those with publications, and decreased to 42.7 ± 48.1 when including all applicants. Percentile analysis revealed that research output was highly right-skewed. For example, an applicant needed to have six publications to be in the 90th percentile of applicants in research output while the 50th percentile had one publication. Lower percentiles (10th-40th) frequently had zero publications or authorships across all categories. This distribution highlights that a minority of students accounted for the majority of publications, indicating a concentrated pattern of research productivity among applicants (Table 2).

**Figure 3:**
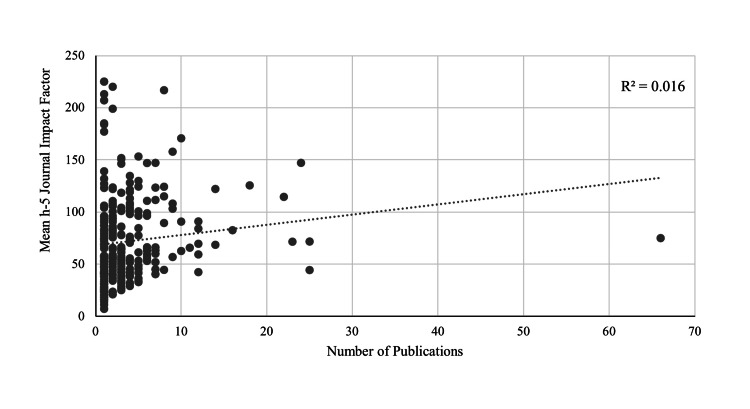
Relationship between the applicant's publication count and mean h-5 journal impact factor Association between the total number of publications per applicant and the applicant’s mean h-5 impact factor, reflecting the relationship between each applicant’s research quantity and journal impact.

Publication metrics of students from NIH T40-funded programs (159/450, 35.3%) were compared with those from programs ranked NIH 41-150 (291/450, 64.7%). Metrics were also compared between male applicants (96/450, 21.3%) and female applicants (351/450, 78.0%). Three students did not have a reported gender and were excluded from the sex-based analyses. Comparisons across total, first-author, top two authors, top five authors, pediatrics-related, basic science, and clinical publications showed no significant differences between T40 and 41-150 (Table 1). There was a slight rank-associated difference in the average journal h-5 index (p=0.044), in which applicants from T40 NIH-funded programs had a higher mean journal h-5 index (NIH T40: 78.2 ± 45.2; NIH 41-150: 67.4 ± 40.0) (Table 1). About 113/159 applicants (71.1%) from T40-funded programs had at least one publication, in contrast to students from NIH 41-150 programs, in which only 157/291 (54.0%) had at least one research item (p<0.001). More than 64% of male students (62/96; 64.6%) had at least one publication, in contrast to 206/351 (58.7%) female students, but this did not yield a statistically significant difference (p=0.296). The same publication metrics used to compare students by NIH rank were used to assess gender-related differences in publication metrics. These comparisons did not yield significant differences based on sex (Table 1). There was a weak correlation (R²=0.016) between the number of research items and the mean h-5 impact factor for each applicant with research (Figure 3).

## Discussion

In this study, we characterized the research output of applicants who successfully matched into the Doximity-ranked top 10 pediatrics residency programs and evaluated differences by gender and NIH funding rank of the medical school. Several notable findings emerged. First, although a majority of applicants had at least one research item, a substantial proportion of matched students (180/450, 40.0%) had no publications at the time of application review. Second, research productivity increased over the course of medical school, particularly for pediatrics-related, first-author, and clinical publications. Third, while applicants from NIH T40-funded medical schools were more likely to have engaged in research, overall publication quantity and authorship metrics did not significantly differ by individual medical school institutional funding rank or gender. Together, these findings suggest that while research involvement is common among matched applicants to highly competitive pediatrics programs, extensive publication records are not uniformly required, and institutional advantages may influence access to research opportunities more than downstream productivity. This is important in the context of increasing total publication counts and author h-index for medical school applicants seeking residency [16].

The average number of publications among matched applicants in our study was modest (Mean=2.3, Median=1.0). This distribution underscores the heterogeneity of research engagement among successful applicants and challenges the notion that high-volume research output is a prerequisite for matching into top pediatrics programs. According to the National Resident Matching Program Charting Outcomes data in 2022, the mean number of abstracts, presentations, and publications for pediatrics-matched U.S. MD senior applicants was 5.6, with a mean of 3.2 research experiences [17]. Our finding of 2.3 mean publications among top-10 program matriculants is notably lower than this national average, which may reflect several factors: (1) timing differences in data collection, (2) our focus on peer-reviewed publications, excluding other scholarly outputs such as abstracts and presentations, or (3) potential variation between top-tier programs and the broader applicant pool. Notably, nearly two-fifths of matched students in our cohort reported no research items, reinforcing the idea that even highly competitive pediatrics programs may value a broader set of applicant attributes, including clinical performance, advocacy, leadership, and commitment to child health, rather than weighing research productivity heavily in the application process [18].

Publications spanned a wide range of journals and article types, with clinical studies representing the largest share. Clinical research was the most common publication type, reflecting both the research infrastructure at most medical schools and the patient-centered nature of pediatrics as a field. The diversity of journals further suggested that applicants were publishing across a broad academic landscape rather than targeting a narrow set of high-impact outlets. Clinical studies were the most numerous publication type for the applicants in our study, which correlates with their ability to be completed remotely and often with a fast turnaround time, which is favorable for students seeking to maximize their research experiences for their residency applications. A plurality of the basic science publications were from before medical school, which is expected as basic science research can require in-person laboratory work and a longer length of time to complete than other types of studies, suggesting that these were completed during college or a post-graduate period. Our study found a trend of increasing total and first author publications over time. This pattern is consistent with increasing exposure to mentors, greater clinical context to guide research interests, and protected research time in later years [19]. The use of a fixed application review date (September 27, 2023) allowed for standardized temporal comparisons. Notably, many of the papers published at the end of the fourth year of medical school could not be included in applicants’ Electronic Residency Application Service (ERAS) applications. Students should be advised to begin conducting research earlier so that their projects can complete the publication process before residency applications are due.

Applicants from NIH Top 40-funded medical schools were significantly more likely to have at least one research item compared to those from schools ranked 41-150. This tracks with previous literature documenting this trend [20]. This finding likely reflects differential access to research infrastructure, funding, mentorship, and institutional culture rather than differences in individual ability or motivation. However, once students had engaged in research, overall publication metrics, including total publications, authorship position, and type of research, did not differ significantly by school funding rank. The absence of differences in most publication metrics suggests that students from less well-funded institutions who do pursue research are able to achieve comparable scholarly output. The modest difference observed in the average journal h-5 index indicates that applicants from highly funded institutions may publish in slightly higher-impact journals, potentially due to institutional prestige, established research pipelines, or mentor networks. Nevertheless, the small effect size suggests that this advantage may be limited in practical significance.

Consistent with national trends in pediatrics, the applicant pool was made up of predominantly female participants, and this gender distribution was statistically significant [21,22]. Despite this imbalance, no gender-related differences were observed across any publication metrics, including total output, authorship position, research type, or journal impact. These findings are encouraging and suggest that, within this highly selected cohort, male and female applicants have comparable research experiences and outputs. This lack of disparity contrasts with reports of gender gaps in academic medicine at later career stages, particularly in senior authorship and leadership roles. Our findings suggest that such disparities may emerge after residency rather than during medical school, highlighting the importance of longitudinal studies tracking academic trajectories beyond the match [23].

Taken together, these results suggest that research engagement is common but not uniformly necessary for matching into top pediatrics residency programs. For applicants, particularly those from institutions with fewer research resources, these findings may offer reassurance that meaningful scholarly engagement, rather than sheer volume, is sufficient, and that lack of publications does not preclude success. For program directors, the wide range of research experiences among matched applicants supports holistic review processes that contextualize research output within institutional opportunities and individual career goals. For policymakers, these findings support investment in research infrastructure at less well-resourced medical schools to reduce access disparities in the residency pipeline.

This study has several notable strengths. First, we examined the complete cohort of all 450 students matching into the top 10 pediatrics programs in 2024, eliminating selection bias inherent to survey-based approaches. Second, our multi-source verification strategy (Google Scholar, social media, program websites) improved attribution accuracy. Third, our temporal analysis linking publications to medical school year provides granular insight into how research productivity evolves across training. Fourth, stratification by both NIH funding rank and gender allows for simultaneous evaluation of two key sources of potential inequity in research access. This study offers a comprehensive examination of research productivity among successfully matched applicants following the shift to pass/fail Step one scoring.

However, several important limitations should be acknowledged. First, our data collection extended six months beyond Match Day, meaning publications appearing between March 15, 2024 and September 27, 2024 could not have influenced match decisions. This may inflate our research metrics relative to what program directors actually reviewed during the application cycle. Second, attribution errors remain possible despite our multi-source verification approach (Scopus, LinkedIn, institutional affiliations), particularly for candidates with common surnames or minimal digital presence. Third, article classification involved some degree of subjective interpretation. Fourth, research output was quantified using publicly available data, which may underestimate scholarly activity such as unpublished work, abstracts, or ongoing projects. Fifth, we recognize that Google Scholar captures only published work and does not account for manuscripts under review, submitted abstracts, or other scholarly activities listed in ERAS. Therefore, our analysis likely underestimates total research engagement. Finally, journal h-5 index, while informative, is an imperfect proxy for research quality and impact.

Our findings reinforce the mounting evidence that residency applications have grown increasingly competitive. The upward trajectory in research productivity among successful applicants persisted through the 2024 Match cycle. Given the recent transition to binary Step one grading, research accomplishments appear to have assumed greater weight in applicant evaluation [19]. We advise prospective candidates to pursue research opportunities as early as feasible to strengthen their applications, though such opportunities in pediatrics remain somewhat limited.

Several important questions remain unanswered and warrant future investigation. First, comparative studies examining applicants who matched versus those who did not match to top programs would help clarify whether research productivity meaningfully differentiates between competitive candidates. Second, longitudinal tracking of these cohorts into fellowship and faculty positions would illuminate whether early research engagement predicts sustained academic productivity. Third, future studies should examine whether the trends identified here are specific to 2024 or reflect a durable post-pass/fail shift in selection criteria. Finally, qualitative research exploring program directors’ actual decision-making processes with respect to research in pediatrics applications would provide important insight into how these metrics are weighted in practice.

## Conclusions

Applicants matching into top pediatrics residency programs in 2024 demonstrated remarkably diverse research backgrounds. While more than half engaged in scholarly work, more than one-third matched successfully with zero publications, challenging assumptions about research as a prerequisite for competitive pediatrics programs. Among those who published, output was modest and highly skewed, with a small minority accounting for most scholarly productivity. Students from highly NIH-funded institutions were more likely to engage in research, yet publication quality and quantity did not differ by institutional rank or gender once students had accessed research opportunities. These findings validate holistic applicant evaluation in pediatrics and suggest that in the post-pass/fail Step one era, top programs may continue to prioritize clinical excellence, commitment to child health, and diverse experiences over research volume. For applicants, particularly those from less-resourced institutions, these results offer reassurance: meaningful engagement and not prolific publication records suffice for success in pediatrics residency matching.
